# Nucleolar Localization of GLTSCR2/PICT-1 Is Mediated by Multiple Unique Nucleolar Localization Sequences

**DOI:** 10.1371/journal.pone.0030825

**Published:** 2012-01-23

**Authors:** Inna Kalt, Ayelet Levy, Tatyana Borodianskiy-Shteinberg, Ronit Sarid

**Affiliations:** The Mina and Everard Goodman Faculty of Life Sciences, Bar Ilan University, Ramat-Gan, Israel; University Hospital of Modena and Reggio Emilia, Italy

## Abstract

The human glioma tumor suppressor candidate region 2 gene product, GLTSCR2, also called ‘protein interacting with carboxyl terminus 1’ (PICT-1), has been implicated in the regulation of two major tumor suppressor proteins, PTEN and p53, and reported to bind the membrane-cytoskeleton regulator of cell signaling, Merlin. PICT-1 is a nucleolar protein, conserved among eukaryotes, and its yeast homolog has been functionally associated with ribosomal RNA processing. By means of confocal microscopy of EGFP and myc-tagged PICT-1 fusion proteins, we delineate that the nucleolar localization of PICT-1 is mediated by two independent nucleolar localization sequences (NoLS). Unlike most NoLSs, these NoLSs are relatively long with flexible boundaries and contain arginine and leucine clusters. In addition, we show that PICT-1 exhibits a nucleolar distribution similar to proteins involved in ribosomal RNA processing, yet does not colocalize precisely with either UBF1 or Fibrillarin under normal or stressed conditions. Identification of the precise location of PICT-1 and the signals that mediate its nucleolar localization is an important step towards advancing our understanding of the demonstrated influence of this protein on cell fate and tumorigenesis.

## Introduction

The human glioma tumor suppressor candidate region 2 gene product GLTSCR2, also called ‘protein interacting with carboxyl terminus 1’ (PICT-1), was initially identified as a 60 kDa (p60) protein partner of two viral proteins, ICP0 and ICP22, encoded by the herpes simplex virus type 1 [1]. The PICT-1 gene (*GLTSCR2*) is located on chromosome 19q within a tumor suppressor region that is frequently lost in human cancers, particularly in gliomas [2]. Accordingly, decreased mRNA and protein expression of PICT-1 is observed in glioblastomas and correlates positively with histological malignant progression [3].

Several studies implicate PICT-1 in the regulation of tumor development and proliferation, but the molecular pathways in which PICT-1 participates are just beginning to be elucidated. PICT-1 interacts with the carboxy-terminus region of the tumor suppressor phosphatase and tensin homolog (PTEN), and appears to promote PTEN phosphorylation and stability [4]. Indeed, knockdown of PICT-1 induces anchorage-independent tumor cell growth and decreases susceptibility to apoptotic death stimuli, whereas overexpression of PICT-1 stimulates caspase and mitochondria-independent cell death; both phenomena reported to be PTEN-dependent [4]–[6]. Given this involvement of PICT-1 in maintaining PTEN stability, it is expected that loss of function mutations in PICT-1 should result in reduced cellular PTEN levels and concomitant deregulated PI3K-mediated signaling. In addition to interacting with PTEN, PICT-1 has been reported to bind the candidate tumor suppressor Moesin-ezrin-radixin-like protein (Merlin) [7]. Expression of PICT-1 was shown to induce translocation of Merlin into the nucleolus and the growth impairment induced by ectopic expression of PICT-1 has been suggested to involve Merlin [7]. Finally, studies using PICT-1 knock-down cells indicate a role in the regulation of DNA damage responses and sensitization to DNA damage [8]. Of note, a recent study challenges the prevailing view of PICT-1, suggesting that PICT-1 is a potentially oncogenic regulator of the MDM2-p53 pathway and acts as a tumor suppressor only under certain conditions, such as loss of p53 function [9]. In accord with this alternative understanding of PICT-1 function, low expression of PICT-1 in colorectal and esophageal cancers bearing intact p53 was found to correlate with increased survival [9].

Recently, we discovered that PICT-1 interacts with the Bcl-2 homolog (KS-Bcl-2) encoded by Kaposi's sarcoma-associated herpesvirus (KSHV) and determined that this interaction selectively relocates KS-Bcl-2 from the mitochondria to the nucleolus. In line with a previous report describing the localization of PICT-1 as a “discrete globular localization pattern” [6], and with data from human nucleolus proteome projects [10]–[12], we evidenced that PICT-1 is located in the nucleolus.

In eukaryotic cells proteins are targeted to the nucleus through the nuclear pore complex (NPC). Small molecules can move freely from the cytoplasm through the NPC, but transport of proteins larger than ∼20 kDa is mediated by specific amino acid sequences, referred to as nuclear localization signal (NLS). A classic NLS contains a cluster of basic amino acids, typically composed of lysines (K) or arginines (R), organized in either a single stretch, the monopartite NLS ((K/R)4–6), or in two stretches, the bipartite NLS, where two small clusters are separated by a few amino acids ((K/R)2X10-12(K/R)3) [13]. A tripartite NLS, comprising three clusters of two or three continuous basic amino acid residues separated by two spacer peptides, has also been described [14]–[16]. Unlike the nucleus, the nucleolus is a membrane-free nuclear structure. Nucleolar localization of proteins is mediated typically by functional interaction with nucleolar core components, such as ribosomal DNA, RNA and proteins, yet in most cases depends on nucleolar localization sequences (NoLS), that often contain clusters of basic amino acids that diverge in context and length [17], [18].

Here, to advance our understanding of the cellular role of PICT-1, we investigate how PICT-1 is targeted to the nucleolus. The sequences involved in nucleolar targeting were mapped by analyzing the intracellular distribution of a panel of myc and EGFP fusion proteins bearing different PICT-1 regions and substitutions. We uncover two unusual, relatively long NoLSs critical for nucleolar targeting of PICT-1. We have also examined the precise nucleolar localization of PICT-1 under normal and stress conditions. Identification of the signals that mediate the cellular localization of PICT-1 is crucial in furthering our understanding of how this protein might function both ordinarily, under stress and during tumorigenesis.

## Results

### Sequence analysis of PICT-1

**Figure 1:**
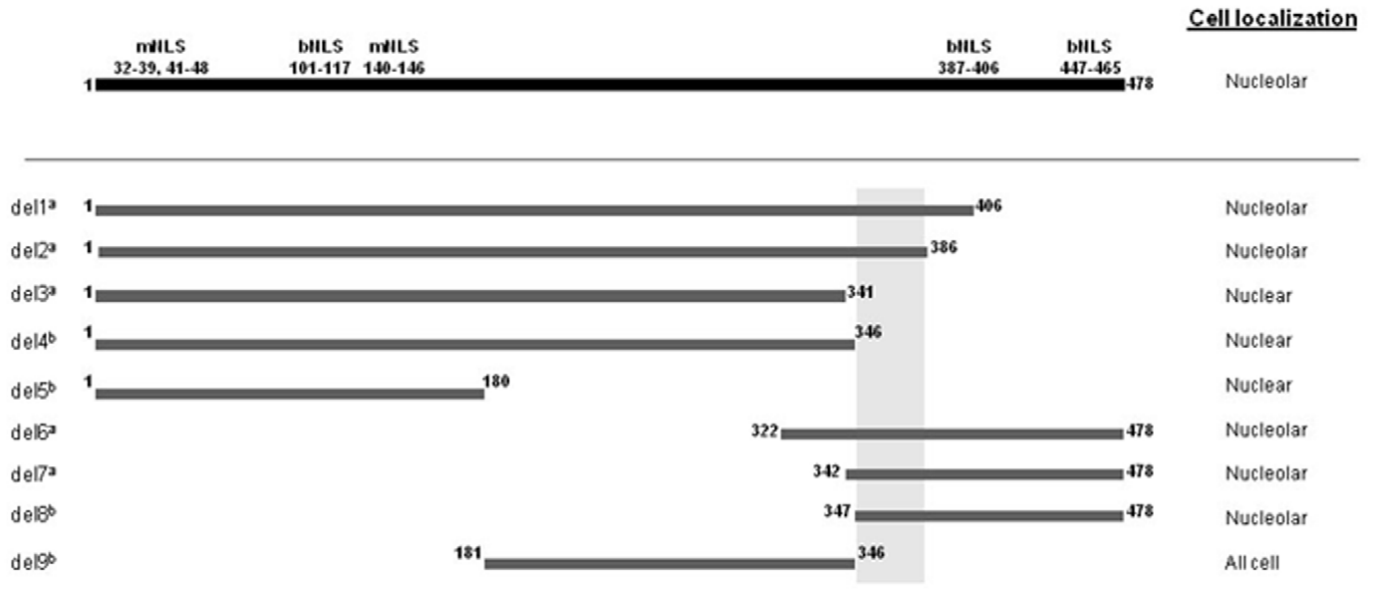
Schematic representation of PICT-1 with its predicted monopartite (m) and bipartite (b) NLSs. Previously reported truncation mutants are shown with their cellular localization indicated on the right, ^a^ refers to [19] and ^b^ refers to [6]. Shadowing denotes the putative nucleolar targeting sequence (aa 347–386) inferred from the localization of these mutants.

PICT-1 comprises 478 amino acids and is reported to contain at least two functional nuclear localization sequences (NLS), at its amino and carboxy termini [6]. Previously, we evidenced that PICT-1 is localized in the nucleolus and predicted the amino acids required for this targeting [19]. We performed a more detailed examination of PICT-1's amino acid sequence using PSORT II, ELM and Nuclear Protein Database tools, which led us to refine our prediction as depicted schematically in Fig. 1: three bipartite NLSs, spanning amino acids 101–117, 387/9/90–405/6 and 447–464/5; and three monopartite NLSs, spanning amino acids 32–39, 41–48, 140–146. In addition, our analysis highlighted scattered short arginine and/or lysine clusters, some of which encompass segments of predicted bipartite NLSs, as possible players in nuclear targeting. Notably, analysis of the amino acid composition of PICT-1 revealed a relatively high content of arginine residues (12.76%) as compared with the general frequency of this residue in vertebrate proteins.

### Characterization of unique motifs in PICT-1 that direct nuclear and nucleolar targeting

As mentioned, previously we expressed ectopically a series of myc-tagged PICT-1 truncation mutants and examined their cellular localization. In light of these earlier results [19] and data from others who tagged PICT-1 with EGFP at the amino terminus [6], we concluded that amino acids 347–386 potentially contain a functional nucleolar localization sequence (NoLS) (Fig. 1). Of note, we observed that EGFP-PICT-1, PICT-1-EGFP, HA-PICT-1, PICT-1-HA and myc-PICT-1 display identical nucleolar distribution patterns (data not shown). Therefore, from this point on we only examine the localization of N-terminally tagged PICT-1 constructs.

**Figure 2:**
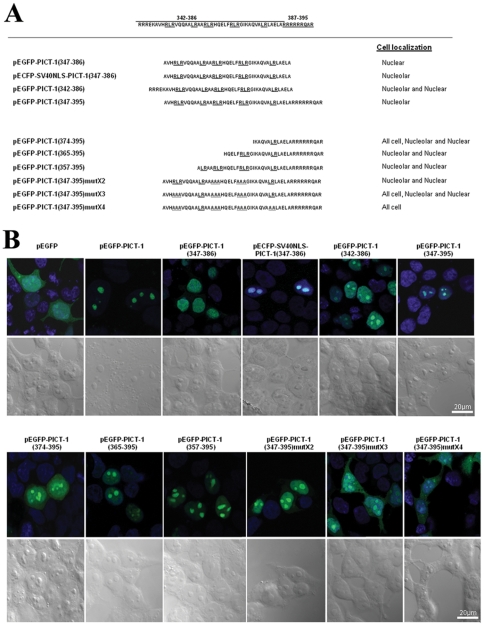
Amino acids 347–395 contain a functional NoLS. (A) The sequences of PICT-1 truncations and point mutants fused to the 3′-end of EGFP are shown. Solid lines highlight RLR and LR sequences whereas dotted lines represent alanine substituted residues. Cellular localization as determined by confocal microscopy is indicated on the right. (B) Confocal microscopic analysis of EGFP fusion proteins. Hoechst stained DNA appears blue. Corresponding differential interference contrast (DIC) images are also shown.

To investigate the ability of amino acids 347–386 to mediate nucleolar targeting, these residues were fused downstream to EGFP (pEGFP-PICT-1(347–386)), the construct expressed in HEK-293T cells and the localization of this fusion protein examined microscopically. In parallel, since the 347–386 amino acid segment does not appear to contain an NLS motif, we constructed and analyzed the localization of other fusion proteins, including one in which we cloned amino acids 347–386 downstream to three copies of the nuclear targeting sequence from SV40 large T Ag (DPKKKRKV) (pECFP-SV40NLS-PICT-1(347–386)), and two others that contained additional flanking arginine-rich PICT-1 amino acids: 342–386 (pEGFP-PICT-1(342–386)) and 347–395 (pEGFP-PICT-1(347–395)) (Fig. 2A). Initially we confirmed by Western analysis using anti-GFP antibody that HEK-293T cells transfected with each plasmid do indeed express fusion proteins matching the predicted sizes (Fig. S1). Subsequent microscopic analysis revealed that amino acids 347–386 failed to target EGFP to the nucleolus, and unexpectedly targeted EGFP to the nucleus, suggesting the presence of a non-canonical NLS in this region (Figs. 2A and B). However, the fusion containing amino acids 347–386 downstream to the known SV40 NLS sequence (pECFP-SV40NLS-PICT-1(347–386)) did indeed exhibit nucleolar EGFP. Moreover, the additional flanking arginine-rich amino acids were observed to facilitate targeting of EGFP to the nucleolus. In the case of the fusion protein containing 387–395 (RRRRRRQAR; pEGFP-PICT-1(347–395)), EGFP was completely nucleolar, whereas the fusion protein containing amino acids 342–346 (RRRER; pEGFP-PICT-1(342–386)) was found to display partial nucleolar localization. Summarily these results, along with previous data concerning truncated mutants of PICT-1, support that amino acids 347–386 contain a functional non-canonical NLS. However, in addition, our findings reveal that amino acids 347–386 fused with a classic monopartite NLS or with a short arginine-rich sequence serve as a functional nucleolar targeting sequence.

Several known functional NLS and NoLS are rich in arginine and/or lysine residues [18]. Upon inspection of the PICT-1 amino acid 347–386 sequence, we identified three RLR motifs and two LR motifs and reasoned that these sequences could participate in nucleolar targeting of PICT-1. To investigate which amino acid sequences are essential for nucleolar targeting, we generated various truncations and mutations in this region and fused each to EGFP: the truncations are pEGFP-PICT-1(374–395), pEGFP-PICT-1(365–395), pEGFP-PICT-1(357–395) and the alanine substitutions are pEGFP-PICT-1(347–395)mutX2, pEGFP-PICT-1(347–395)mutX3, and pRS443/pEGFP-PICT-1(347–395)mutX4) (Fig. 2A). As before, each plasmid was confirmed to express the appropriately sized protein using Western analysis of transiently transfected HEK-293T cells (Fig. S1). Microscopic analysis of the panel of fusion proteins highlighted the importance of the entire 347–395 amino-acid sequence for nucleolar localization. More extensive truncations in the PICT-1 region resulted in EGFP displaying decreased levels of nucleolar localization, along with increased nuclear background staining. Indeed, confocal imaging of the alanine substitution mutants corroborated that the entire sequence is necessary for efficient nucleolar targeting and, in particular that the RL motifs play a role in nucleolar targeting. Accordingly, when EGFP was fused to a 347–395 PICT-1 amino acid region in which the three RLR motifs and one LR motif had been substituted to alanine, the fusion protein failed to localize to the nucleus or the nucleolus, supporting that both an NLS and NoLS are contained within these amino acids.

In summary, our findings evidence a unique sequence within amino acids 347–386 that can operate together with adjacent N or C-terminal arginine-rich sequences to target proteins to the nucleolus.

### Identification of an additional functional NoLS motif in PICT-1

**Figure 3:**
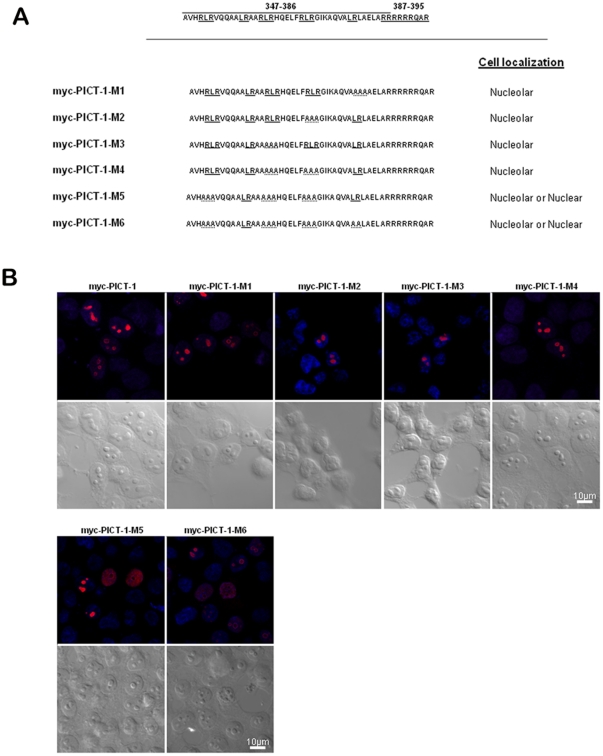
PICT-1 substitution mutants within the NoLS spanning amino-acids 347–395 retain the ability to be targeted to the nucleolus. (A) The sequences of myc-tagged full-length PICT-1 substitution mutants (M1–M6) are shown. Mutated residues are indicated by dotted lines. Cellular localization as determined by confocal microscopy is indicated on the right. (B) Confocal microscopic analysis of fusion proteins. 24 hrs after transfection, cells were stained with anti-myc antibody and rhodamine-conjugated secondary antibody. Hoechst stained DNA appears blue. Corresponding differential interference contrast (DIC) images are also shown.

In light of the results described above, we predicted that deleting key amino acids in the 347–386 PICT-1 region in the context of full length myc-tagged PICT-1 would impair nucleolar targeting. We constructed a series of myc-tagged full length PICT-1 substitution mutants (M1–M6, Fig. 3A), confirmed expression of appropriately sized proteins and then examined the localization of each mutant with anti-myc antibodies (Fig. S2). No nuclear staining was evident in cells transfected with a control vector. Surprisingly, the M1–M4 mutants were observed to exhibit nucleolar localization similar to wild type myc-PICT-1, and only the M5 and M6 mutants demonstrated a different and unusual pattern. Each were found either in the nucleus or in the nucleolus, two mutually exclusive localization phenotypes within one cell population. Overall, these observations indicated that PICT-1 contains another NoLS.

**Figure 4:**
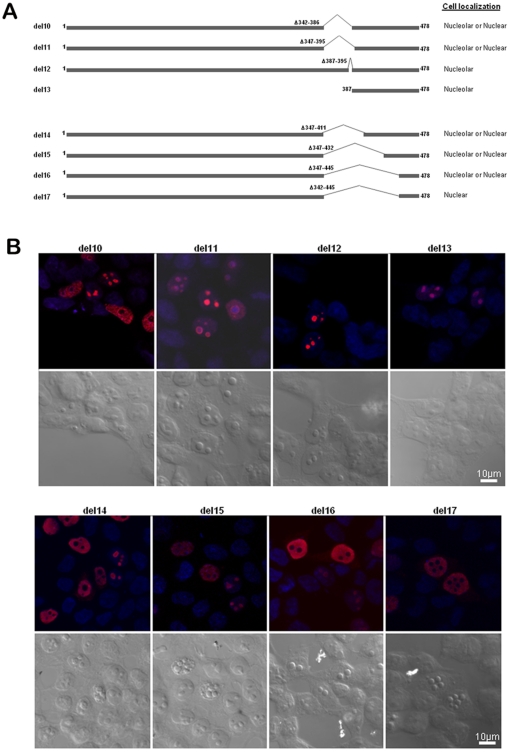
Amino acids 387–478 contain a functional NoLS. (A) The sequences of myc-tagged PICT-1 deletion mutants are shown. Cellular localization as determined by confocal microscopy is indicated on the right. (B) Confocal microscopic analysis of fusion proteins. 24 hrs after transfection, cells were stained with anti-myc antibody and rhodamine-conjugated secondary antibody. Hoechst stained DNA appears blue. Corresponding differential interference contrast (DIC) images are also shown.

To corroborate this assertion, we constructed additional internal deletion mutants of PICT-1 (Fig. 4), confirmed expression upon transfection into HEK-293T cells and examined their cellular localization (Fig. S3). The PICT-1 mutant lacking amino acids 387–395 (del12) still localized to the nucleolus, supporting our premise that there is an additional NoLS somewhere else in the protein (Fig. 4). However, when we examined in more detail the localization of ectopically expressed PICT-1 mutants lacking amino acids 342–386 (del10) or 347–395 (del11), we noted that approximately 50% of the cells exhibit nuclear PICT-1 and 50% nucleolar PICT-1, supporting that this region does indeed have a role in nucleolar targeting, though not an exclusive one. One interpretation of these findings is that deleting the amino acids 347–395 alters the conformation of PICT-1 and affects the accessibility of a second putative NoLS to interacting molecules that target the protein the nucleolus. Moreover, post-translational modifications that take place under certain conditions could alter the protein conformation and hence affect the accessibility to its second NoLS. The finding that a PICT-1 truncation mutant containing only amino acids 387–478 (del13) accumulates in the nucleolus confirmed that PICT-1 does indeed contain a second functional NoLS that can independently target PICT-1 to the nucleolus.

**Figure 5:**
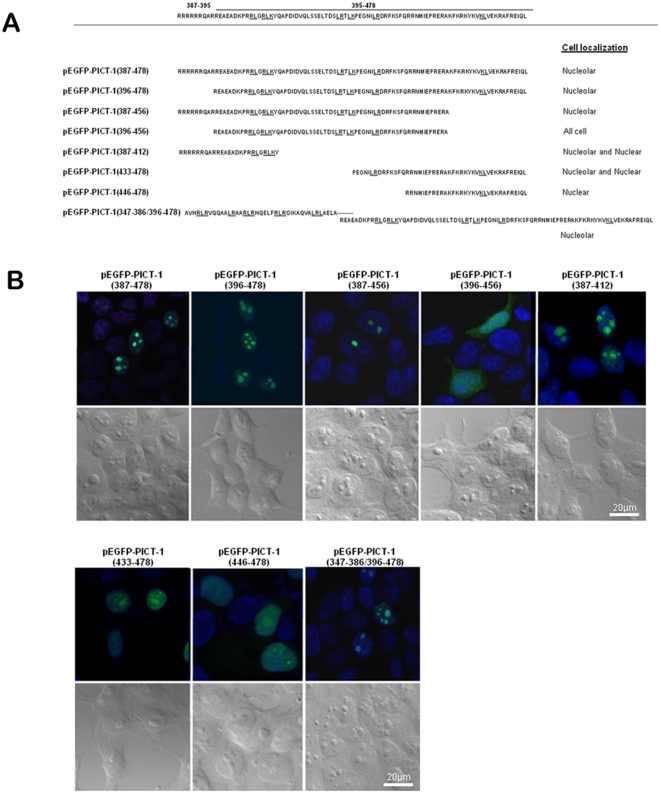
Amino acids 387–478 contain a functional NoLS that can target EGFP to the nucleolus. (A) PICT-1 sequences fused to EGFP are shown. Solid lines highlight LR/RL and KL/LK sequences. Cellular localization as determined by confocal microscopy is indicated on the right. (B) Confocal microscopic analysis of EGFP fusion proteins. Images were taken 24 hrs after transfection. Hoechst stained DNA appears blue. Corresponding differential interference contrast (DIC) images are also shown.

Two potential bipartite NLS motifs spanning amino acids 387/9/90–405/6 and 447–464/5 were predicted within the amino acid sequence 387–478. To precisely map the essential amino acids within this region required for nucleolar targeting of PICT-1, we constructed more EGFP fusion proteins (Fig. 5A, pEGFP-PICT-1(387–478), pEGFP-PICT-1(396–478), pEGFP-PICT-1(387–456), pEGFP-PICT-1(396–456), pEGFP-PICT-1(387–412), pEGFP-PICT-1(433–478) and pEGFP-PICT-1(446–478)). As before, each construct was demonstrated to express a protein of the expected size before its cellular localization was analyzed microscopically (Fig. S4). In accord with the localization of the myc-tagged PICT-1 truncation mutant, EGFP fused to amino acids 387–478 exhibited nucleolar accumulation. Further truncations of this 387–478 segment from its N-terminus were observed to gradually reduce nucleolar localization. Notably, EGFP fused to amino acids 347–478 but lacking the arginine-rich amino acids 387–395 (pRS455/pEGFP-PICT-1(347–386/396–478)) was still localized to the nucleolus. This latter finding corroborates our observation that myc-tagged PICT-1 mutants with deletions spanning 347–411 (del14), 347–432 (del15) or 347–445 (del16) exhibit two mutually exclusive phenotypes, whereby the protein is either located in the nucleus or in the nucleolus. A myc-tagged PICT-1 mutant with a larger deletion spanning 342–445 (del17) displayed only nuclear localization with no nucleolar localization at all (Fig. 4).

Taking all the data together, we conclude that PICT-1 contains two functional NoLSs, between amino acids 347–395 and 396–478. Both NoLSs encompass pairs or triplets of adjacent arginine/lysine-leucine residues and possess flexible boundaries, such that amino acids 342–386 or 387–456 still direct nucleolar targeting, though with increased nuclear background localization. Notably, our mapping results indicate that, unlike classical nucleolar localization signals, the nucleolar targeting motifs of PICT-1 are characterized by relatively long sequences containing arginine/lysine-leucine motifs.

### Nucleolar localization of endogenous PICT-1

**Figure 6:**
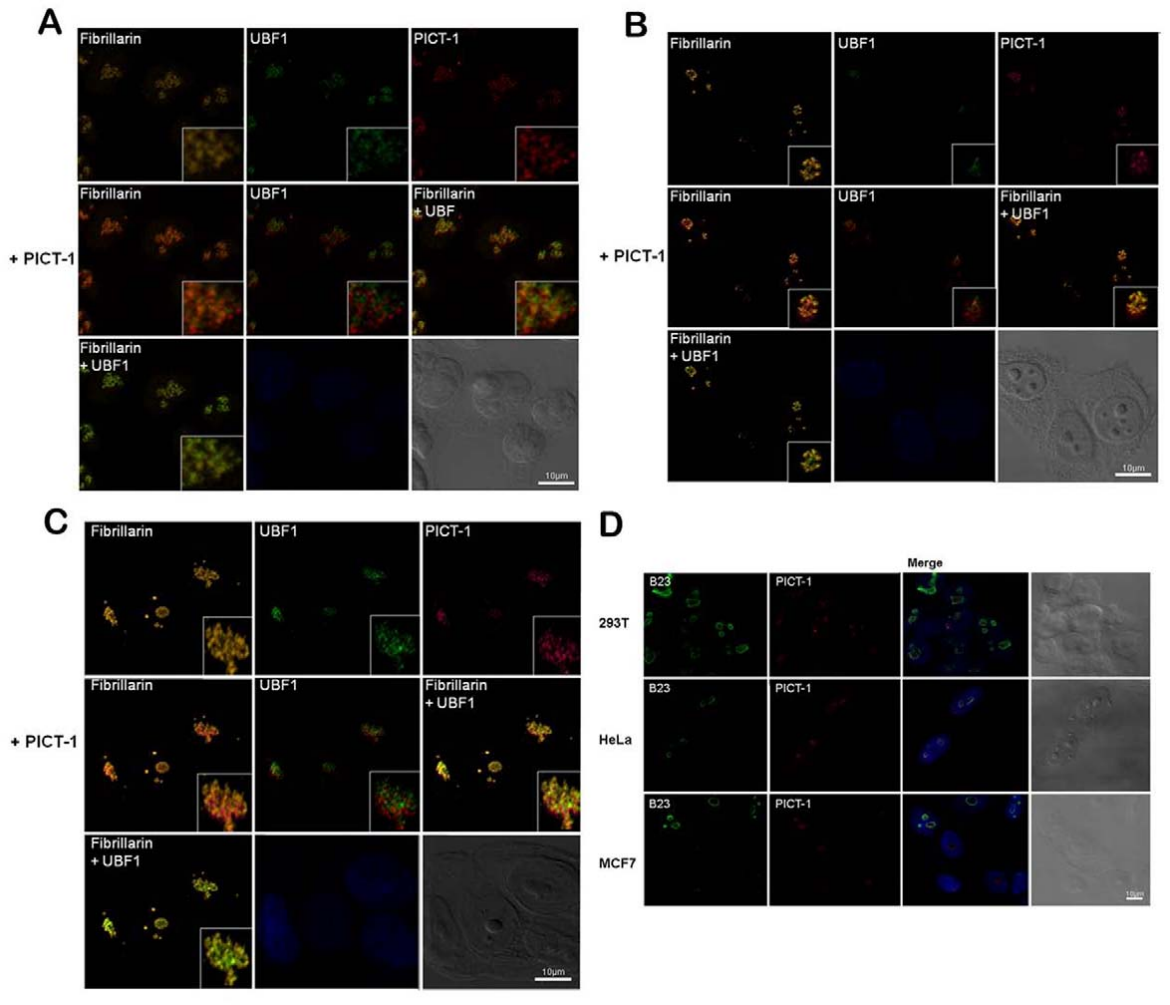
Nucleolar localization of PICT-1 in (A) HEK-293T, (B) HeLa and (C) MCF7 cells. Fibrillarin, UBF1 and PICT-1 were stained using Cy5 (orange), FITC (green) and Cy3 (red)-coupled secondary antibodies, respectively. Where indicated, merged images are shown. Nuclei were stained with Hoechst. (D) B23 and PICT-1 were double stained using FITC and Cy3 secondary antibodies, respectively, in HEK-293T, HeLa and MCF7 cells.

The nucleolus is a complex cluster of molecules organized in a defined manner, with an architecture characterized by the fibrillar center (FC) and the dense fibrillar (DFC) and granular (GC) components [20]. To determine the precise nucleolar location where PICT-1 resides, we performed colocalization experiments using confocal microscopy and known nucleolar markers. Nucleoli were also visualized using differential interference contrast, where they appear as elevated regions within the nucleus. PICT-1 was observed to exhibit a nucleolar pattern reminiscent of upstream binding factor 1 (UBF1) and Fibrillarin, each of which typically localize to the FC and DFC compartments, respectively. A similar protein distribution was evident in HEK-293T, HeLa and MCF-7 cells. However, PICT-1 was not found to co-localize with endogenous Fibrillarin or UBF1 (Fig. 6). Similar results were obtained upon switching between the secondary antibody employed (data not shown). As expected from the apparent nucleolar distribution of PICT-1, PICT-1 also did not co-localize with the GC protein B23.

### Determination of PICT-1 localization upon stress

**Figure 7:**
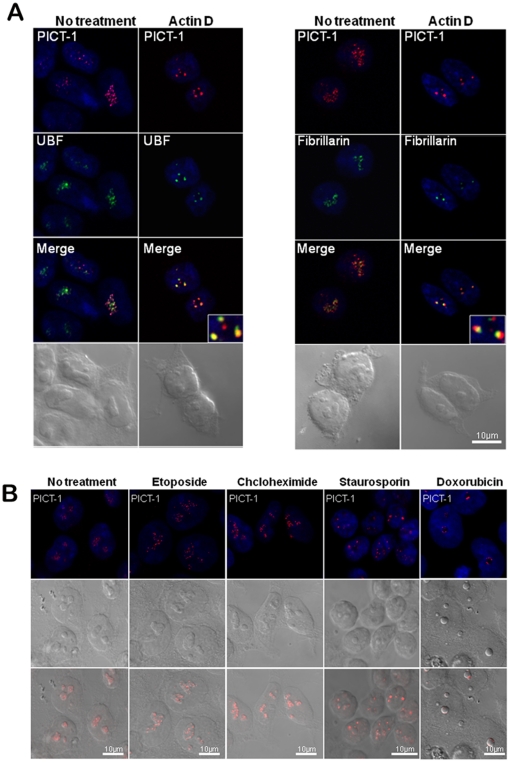
Cellular localization of endogenous PICT-1 upon stress induction. (A) Nucleolar segregation was induced by actinomycin D treatment (0.05 µg/ml) of HEK-293T for 4 hrs. Fibrillarin, UBF1 and PICT-1 were stained using Cy5 (orange), FITC (green) and Cy3 (red)-coupled secondary antibodies, respectively. Where indicated, merged images are shown. Nuclei were stained with Hoechst. Some PICT-1 is found in Fibrillarin containing nucleolar caps and to a greater extent in caps containing UBF1. (B) HEK-293T cells were treated with etoposide, cycloheximide, staurosporine or doxorubicin, for 24, 1, 24 and 2 hrs, respectively. Cells were then fixed and stained with anti PICT-1 and rhodamineconjugated secondary antibody.

In some studies, induction of nucleolar segregation or cell stress clarified the association of nucleolar proteins with their exact nucleolar compartment. Therefore, we induced nucleolar segregation by treatment with low levels of the RNA polymerase I inhibitor Actinomycin D (50 ng/ml). This treatment triggered the appearance of PICT-1 in cap structures, situated at the external part of the nucleolus, and this location of PICT-1 was found to partially overlap the cap structures containing endogenous Fibrillarin and UBF1 (Fig. 7A). A similar localization was observed upon DNA damage caused by doxorubicin treatment, a known inducer of nucleolar stress, which causes the release of ribosomal proteins into the nucleoplasm and activates p53. Notably, translation inhibition by cycloheximide and topoisomerase II inhibition by etoposide did not abrogate nucleolar localization and distribution of PICT-1, though kinase inhibition by staurosporin did, such that PICT-1 became localized to nuclear dots in the nucleoplasm (Fig. 7B).

## Discussion

Detailed analyses led us to predict that PICT-1 contains six NLS motifs as well as several short arginine and lysine-rich clusters, the latter sharing similarity with functional NoLSs described in nucleolar proteins. In the present study, we evidence a non-canonical NLS sequence spanning amino acids 347–386 of PICT-1. This region is arginine-rich and contains 22 hydrophobic non-arginine amino acids out of 32, and thus is similar to other functional NLS motifs composed of positively charged amino acids located within a hydrophobic stretch [21]. For example, the nuclear histone deacetylase 4 (HDAC4) protein was shown to contain a similar functional NLS, containing three arginine/lysine clusters among leucine residues [15] and the bovine immunodeficiency virus Rev protein contains a bipartite NLS with long spacer region [22]. Although unlikely, it is possible that an as yet unknown protein interacts with this non-canonical NLS of PICT-1, regulating subcellular localization [15].

The major function of the nucleolus is to coordinate the assembly of ribosomal subunits, a complex process involving the synthesis and processing of rRNA and the production of pre-ribosomal particles. However, the nucleolus also plays important roles in cell cycle control, tumorigenesis, viral replication, senescence and stress responses and hence contains proteins not associated, at least directly, with the biogenesis of ribosomal subunits [12], [20], [23]–[25]. Unlike the active transport mechanisms characterized for nuclear localization, nucleolar localization does not appear to involve a universal mechanism and instead is mediated by various interactions with nucleolar components. Accordingly, nucleolar localization sequences (NoLS) represent domains that interact with resident ribosomal components and other resident proteins. Most experimentally validated NoLSs are rich in arginine and lysine residues and are localized in easily accessible alpha-helices or coils at the surface of proteins [17], [18]. Bipartite and tripartite NoLS, composed of two or three clusters of basic amino acids or NLSs, have been described as well [26], [27]. Here, we identify two amino acid motifs, with flexible boundaries, that can independently target PICT-1 to the nucleolus. To date, several proteins have been reported to contain multiple NoLS motifs [27]. In the case of PICT-1, the NoLS motifs require a nearby NLS or arginine-rich cluster to function, such that only when the long sequence is fused to an unrelated protein, like EGFP, is nucleolar targeting observed. Thus, unlike several experimentally validated nucleolar targeting signals composed primarily of short stretches of basic amino acids, the nucleolar targeting motif of PICT-1 encompasses a much longer sequence. Other proteins containing two NLSs where one directs entry into the nucleus and the other targets the protein to the nucleolus, such as Cirhin, the protein phosphatase-1 inhibitor-3 (PPP1R11) and the phosphatidylinositol 4-kinase PI4K230, have been shown to possess similarly complex nucleolar targeting motifs [28]–[30]. More specifically, as we evidenced for PICT-1, two leucine residues within the relatively long Cirhin putative NoLS are critical for nucleolar targeting [30]. Of note, an artificial neural network recently developed to identify NoLS motifs predicted three motifs spanning amino acids 26–57, 90–117 and 450–476 of PICT-1 [18]. However, we establish here that although all three predicted motifs can function as NLSs, none can mediate targeting of PICT-1 to the nucleolus thus strengthening the uniqueness of our findings.

Predictions of PICT-1 structure using the XtalPred tool [31] indicate large disordered regions, suggesting that this protein could assume distinct protein confirmations upon different post-translational modifications or protein-protein interactions. Given that an NoLS motif must be present at the protein surface to interact with the relevant partner bringing it to the nucleolus, the efficacy of each NoLS in the context of the full length protein could depend on post-translational events. In other words, multiple NoLS motifs could function to secure nucleolar localization of PICT-1 in different conditions. In support of this hypothesis, we observed that transfection of the M5, M6, del10, del11, del14, del15 and del16 myc-PICT-1 mutants resulted in two distinct PICT-1 expressing phenotypes, one with nuclear PICT-1 and the other with nucleolar PICT-1 (Fig. 4). This observation has been repeatedly obtained, both in HEK-293T and HeLa cells, and was not affected by transfection efficiency. Each of these PICT-1 mutants lacks the upstream NoLS but contain the second functional NoLS near the carboxy terminus. Since fusion of only the second carboxy terminal NoLS to the 3′-end of EGFP directs uniform nucleolar localization (Fig. 5), we suspect that structural restrictions influence the dichotomous localization of these PICT-1 mutants and likely also play a role in localization of the wild type full length protein.

As mentioned above, the nucleolus is a highly organized nuclear sub-compartment with characteristic functional architecture, the fibrillar center (FC) and the dense fibrillar (DFC) and granular (GC) components. rRNA genes are clustered mostly in the FC along with RNA polymerase I, topoisomerase I and UBF1. Processing, cleavage, modifications and maturation of pre-rRNA take place in the DFC and involve proteins such as Fibrillarin. Certain processing events likely occur also in the GC, where mature rRNA and ribosomal proteins are assembled into the large and small ribosomal subunits. The FC-DFC boundary is the predominant site of pre-rRNA transcription by RNA polymerase I [20], [32]. We observed a characteristic dotted nucleolar pattern of PICT-1 staining in multiple cell types. This staining pattern resembled those of UBF1 and Fibrillarin, yet PICT-1 was not found to colocalize with these proteins under unstressed conditions. Nevertheless, PICT-1 was observed to imitate the relocation of Fibrillarin and UBF1 into nucleolar caps demonstrated previously to occur in response to transcription stress, such as conditions that inhibit RNA Pol I (low levels actinomycin D) [33]. Nucleolar caps tend to preserve preexisting protein interactions, but can enable the establishment of new interactions [11], [33]. Our finding that PICT-1 exhibits comparable, yet not identical, nucleolar distribution and segregation as Fibrillarin and UBF1 leads us to speculate that PICT-1 is involved somehow in regulating the rRNA biosynthesis machinery, occupying a special site near the rDNA areas. Yet, as PICT-1 contains no obvious rRNA/RNA binding motif, it is unlikely to be directly involved in rRNA biogenesis. We found that treatment with doxorubicin, a topoisomerase II inhibitor, triggered peripheral nucleolar localization of PICT-1, whereas treatment with staurosporin, a general kinase inhibitor, resulted in the release of PICT-1 from the nucleolus. The latter observation could be interpreted as indicating that PICT-1 localization depends on phosphorylation, either of itself or a cellular partner.

PICT-1 is conserved among eukaryotes and inactivation of the yeast ortholog, NOP53p, is lethal or impairs cell growth. Nop53p participates in ribosome biogenesis and is required for late steps in the processing of the large ribosomal subunit RNAs by the exosome [34]–[36]. Knock-down of the Caenorhabditis elegans PICT-1 homologue (Y39B6A) results in developmental defects (slow growth and larval arrest) [37], [38]. A role for human PICT-1 in ribosome biosynthesis is supported by its nucleolar localization [10], [11], but has not been evidenced directly. PICT-1 is required for the survival and proliferation of embryonic stem cells and is essential for preimplantation embryogenesis; mice bearing a null mutation in *pict-1* do not develop [9]. This study, identifying the precise cellular localization of PICT-1 and signals influencing its localization, represents an important step towards an understanding of how this protein functions normally and during tumorigenesis

## Materials and Methods

### Chemicals and antibodies

Mouse anti-GFP (Covance Research Products) anti-myc (myc-Tag 9B11) (DSHB), anti-B23/Nucleophosmin (AB10530; Abcam) and anti-UBF (H00007343-M01), goat anti-PICT-1 (C-20, Santa Cruz), and rabbit anti-Fibrillarin (AB5821; Abcam) were used with either horseradish peroxidase-conjugated, rhodamine, FITC, Cy3 or Cy5-conjugated anti-mouse (Jackson ImmunoResearch Laboratories, Inc.), anti-goat or anti-rabbit IgG (Bio-Rad Laboratories). Drugs used: actinomycin D (RNA polymerase I inhibition 0.05 µg/ml, 4 hrs), cycloheximide (100 µM, 1 hr), doxorubicin (10 µM, 2 hrs), etoposide (3 µM, 24 hrs), staurosporine (1 µM, 24 hrs) (Sigma).

### Plasmids

The pEF1-myc-PICT-1 mammalian expression vector was kindly provided by T. Maehama (Tokyo Metropolitan University, Tokyo, Japan) [4]. Plasmids for the expression of N-terminally ECFP and C-terminally EGFP-fused PICT-1 protein (designated as ECFP-PICT-1 and PICT-1-EGFP, respectively) were generated using the full-length PICT-1 plasmid as a template for PCR with forward and reverse primers listed on Table S1. PCR products were then digested and cloned into pECFP-N1 or pEGFP-C2, respectively. Expression plasmids containing myc or EGFP fusion of PICT-1 deletion and substitution mutants were constructed using the full-length PICT-1 plasmid or already mutated PICT-1 plasmids as a template for PCR with two rounds of amplification. During the first round of PCR, we used the flanking outer primers that contained restriction sites, together with overlapping inner sense and antisense primer pairs. This round produced amplification products that overlapped at their 3′ and 5′ ends. A second round amplification used these products as amplification templates with the outer primers. PCR fragments of PICT-1, generated by the second round of PCR, were then digested and inserted into the appropriate vectors.

### Cell culture and transfection

HEK-293T (human epithelial kidney), HeLa (human cervical carcinoma) and MCF7 (human breast adenocarcinoma) cells were obtained from the American Type Culture Collection (ATCC), and grown in Dulbecco's modified Eagle's medium (DMEM) supplemented with 10% fetal calf serum (FCS) (Biological Industries, Kibbutz Beit Haemek, Israel) and antibiotics. All cells were maintained at 37°C in a humidified atmosphere with 5% CO_2_. DNA transfections into cells employed the TransIT-LT1 reagent (Mirus Bio LLC).

### Western blot analysis

Cells were washed twice in cold phosphate-buffered saline (PBS), suspended in RIPA lysis buffer and incubated on ice for 30 min. Then cell debris was removed by centrifugation at 12,000×g for 15 min at 4°C. Protein lysates were resolved by SDS-PAGE and transferred to nitrocellulose membranes (Pall Gelman Laboratory, Germany). The protein contents of different samples were verified by Ponceau S staining. The nitrocellulose membranes were blocked with 5% dry milk in TBS and subsequently incubated with primary antibody. Specific reactive bands were detected using goat anti-rabbit IgG or goat anti-mouse conjugated to horseradish peroxidase. Immunoreactive bands were visualized using the EZ-enhanced chemiluminescence (ECL) detection kit (Biological Industries, Israel).

### Immunofluorescence microscopy

Cells were seeded on coverslips prior to transfection. At 24-hr post transfection, cells were washed with PBS and fixed by incubation in 4% formaldehyde for 20 min at room temperature. The cells were then washed twice in PBS and permeabilized in PBS containing 0.1% Triton ×100 and 1% BSA at room temperature for 30 min. Cells were then probed with primary antibody at 4°C and conjugated secondary antibodies were then applied for detection. To stain the nuclei, the cells were incubated for 30 min with 0.05 µg/ml Hoechst dye (Sigma). Cells were examined and photographed under a confocal laser-scanning microscope (Zeiss LSM 510 META). A similar procedure was taken to detect endogenous proteins.

## Supporting Information

Figure S1 **Amino acids 347–395 contain a functional NoLS.** Western analysis of the expression of EGFP-tagged PICT-1 fusion proteins using anti-GFP antibody.
(TIF)

Figure S2 **PICT-1 substitution mutants within the NoLS spanning amino-acids 347–395 retain the ability to be targeted to the nucleolus.** Western analysis of the expression of myc-tagged PICT-1 substitution mutants using anti-myc antibody.
(TIF)

Figure S3 **Amino acids 387–478 contain a functional NoLS.** Western analysis of the expression of myc-tagged PICT-1 deletion mutants using anti-myc antibody.
(TIF)

Figure S4 **Amino acids 387–478 contain a functional NoLS that can target EGFP to the nucleolus.** Western analysis of the expression of EGFP-tagged PICT-1 fusion proteins using anti-GFP antibody.
(TIF)

Table S1 Primers for constructing plasmids, deletion and substitution mutants.
(DOC)
