# Supplementary figures and images for: Nucleolar Localization of GLTSCR2/PICT-1 Is Mediated by Multiple Unique Nucleolar Localization Sequences
Source: PLoS One. 2012 Jan 23;7(1):e30825. doi: 10.1371/journal.pone.0030825 (PMC3264635; doi:10.1371/journal.pone.0030825)

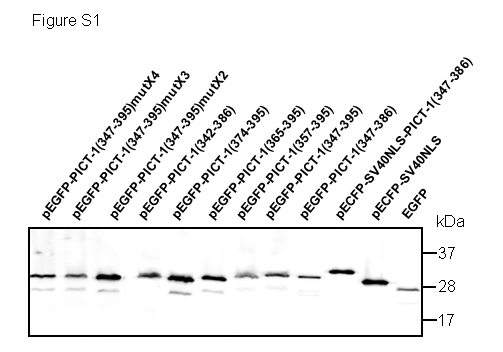

Supplement: Figure S1 — Amino acids 347–395 contain a functional NoLS. Western analysis of the expression of EGFP-tagged PICT-1 fusion proteins using anti-GFP antibody. (TIF) [file pone.0030825.s001.tif]

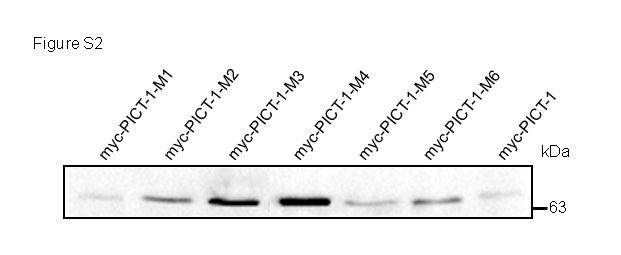

Supplement: Figure S2 — PICT-1 substitution mutants within the NoLS spanning amino-acids 347–395 retain the ability to be targeted to the nucleolus. Western analysis of the expression of myc-tagged PICT-1 substitution mutants using anti-myc antibody. (TIF) [file pone.0030825.s002.tif]

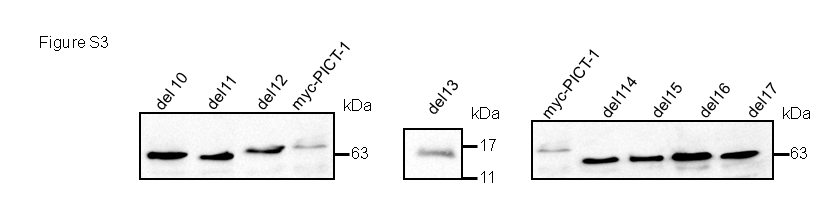

Supplement: Figure S3 — Amino acids 387–478 contain a functional NoLS. Western analysis of the expression of myc-tagged PICT-1 deletion mutants using anti-myc antibody. (TIF) [file pone.0030825.s003.tif]

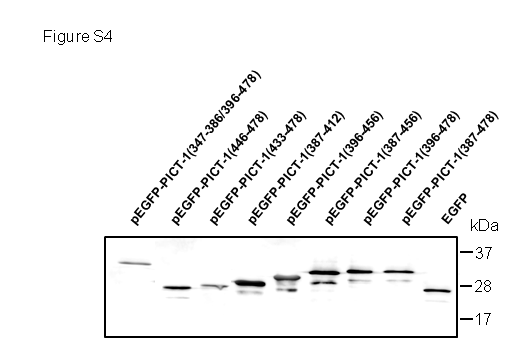

Supplement: Figure S4 — Amino acids 387–478 contain a functional NoLS that can target EGFP to the nucleolus. Western analysis of the expression of EGFP-tagged PICT-1 fusion proteins using anti-GFP antibody. (TIF) [file pone.0030825.s004.tif]
