# Supplementary material for: Nucleolar Localization of GLTSCR2/PICT-1 Is Mediated by Multiple Unique Nucleolar Localization Sequences
Source: PLoS One. 2012 Jan 23;7(1):e30825. doi: 10.1371/journal.pone.0030825 (PMC3264635; doi:10.1371/journal.pone.0030825)
Supplement: Table S1 — Primers for constructing plasmids, deletion and substitution mutants. (DOC) [file pone.0030825.s005.doc]

**Table S1:** Primers for constructing plasmids, deletion and substitution mutants. Restriction enzyme recognition sites are underlined.

| **Plasmid designation** | **Forward primer (5’-3’)** | **Reverse primer (5’-3’)** | **Template** |
| --- | --- | --- | --- |
| ECFP-PICT-1 | AAAGGTACCATGGCGGCAGGA | GCATGGATCCCTACAACTGGATCTCACGGAA | myc-PICT-1 |
| PICT-1-EGFP | AAAGAATTCAGATGGCGGCAGGAGGCA | AAAGGATCCAGCAACTGGATCTCACG | myc-PICT-1 |
| pEGFP-PICT-1(347-386) | AAAGGATCCGCTGTGCACAGGCTGCGG | AAATCTAGAATTCGCCAGCTCCGCCAGCCTCAG | myc-PICT-1 |
| pECFP-SV40NLS-PICT-1(347-386) | AAAGGATCCGCTGTGCACAGGCTGCGG | AAATCTAGAATTCGCCAGCTCCGCCAGCCTCAG | myc-PICT-1 |
| pEGFP-PICT-1(342-386) | AAAGGATCCCGGCGGCGGGAGAAGGCTGTG | AAATCTAGAATTCGCCAGCTCCGCCAGCCTCAG | myc-PICT-1 |
| pEGFP-PICT-1(347-395) | AAAGAATTCGCTGTGCACAGGCTGCGG | TTTGGATCCTTACCGCCGCGCGCCTGCCG | myc-PICT-1 |
| pEGFP-PICT-1(374-395) | AAAGAATTCGGGATCAAGGCCCAGGTG | TTTGGATCCTTACCGCCGCGCGCCTGCCG | myc-PICT-1 |
| pEGFP-PICT-1(365-395) | AAAGAATTCTTGCGGGCCGCCCGGCTCCGG | TTTGGATCCTTACCGCCGCGCGCCTGCCG | myc-PICT-1 |
| pEGFP-PICT-1(357-395) | AAAGAATTCCACCAGGAGCTGTTCCGG | TTTGGATCCTTACCGCCGCGCGCCTGCCG | myc-PICT-1 |
| pEGFP-PICT-1(347-395)mutX2 | AAAGAATTCGCTGTGCACAGGCTGCGG | TTTGGATCCTTACCGCCGCGCGCCTGCCG | myc-PICT-1-M4 |
| pEGFP-PICT-1(347-395)mutX3 | AAAGAATTCGCTGTGCACGCTGCTGCT | TTTGGATCCTTACCGCCGCGCGCCTGCCG | myc-PICT-1-M4 |
| pEGFP-PICT-1(347-395)mutX4 | AAAGAATTCGCTGTGCACGCTGCTGCT | TTTGGATCCTTACCGCCGCGCGCCTGCCG | myc-PICT-1-M6 |
| myc-PICT-1-M1  1^st^ cycle PCR  2^nd^ cycle PCR | CCGGGTACCATGGAACAAAAACTCATCTCA  CAGGTGGCCGCTGCTGCTGCGGAGCTG  CCGGGTACCATGGAACAAAAACTCATCTCA | CAGCTCCGCAGCAGCAGCGGCCACCTG  AACTCTAGAGCAGCTACAACTGGATCTCA  AACTCTAGAGCAGCTACAACTGGATCTCA | myc-PICT-1  myc-PICT-1  1^st^ cycle PCR products |
| myc-PICT-1-M2  1^st^ cycle PCR  2^nd^ cycle PCR | CCGGGTACCATGGAACAAAAACTCATCTCA  CGGGCCGCCGCTGCTGCTCACCAGGAGCTG  CCGGGTACCATGGAACAAAAACTCATCTCA | CAGCTCCTGGTGAGCAGCAGCGGCGGCCCG  AACTCTAGAGCAGCTACAACTGGATCTCA  AACTCTAGAGCAGCTACAACTGGATCTCA | myc-PICT-1  myc-PICT-1  1^st^ cycle PCR products |
| myc-PICT-1-M3  1^st^ cycle PCR  2^nd^ cycle PCR | CCGGGTACCATGGAACAAAAACTCATCTCA  GAGCTGTTCGCTGCTGCTGGGATCAAGGCC  CCGGGTACCATGGAACAAAAACTCATCTCA | GGCCTTGATCCCAGCAGCAGCGAACAGCTC  AACTCTAGAGCAGCTACAACTGGATCTCA  AACTCTAGAGCAGCTACAACTGGATCTCA | myc-PICT-1  myc-PICT-1  1^st^ cycle PCR products |
| myc-PICT-1-M4  1^st^ cycle PCR  2^nd^ cycle PCR | CCGGGTACCATGGAACAAAAACTCATCTCA  GAGCTGTTCGCTGCTGCTGGGATCAAGGCC  CCGGGTACCATGGAACAAAAACTCATCTCA | GGCCTTGATCCCAGCAGCAGCGAACAGCTC  AACTCTAGAGCAGCTACAACTGGATCTCA  AACTCTAGAGCAGCTACAACTGGATCTCA | myc-PICT-1-M2  myc-PICT-1-M2  1^st^ cycle PCR products |
| myc-PICT-1-M5  1^st^ cycle PCR  2^nd^ cycle PCR | GCTGTGCACGCTGCTGCTGTACAGCAGGCC  GCTGTGCACGCTGCTGCTGTACAGCAGGCC  CCGGGTACCATGGAACAAAAACTCATCTCA | GGCCTGCTGTACAGCAGCGTGCACAGC  AACTCTAGAGCAGCTACAACTGGATCTCA  AACTCTAGAGCAGCTACAACTGGATCTCA | myc-PICT-1  myc-PICT-1-M4  1^st^ cycle PCR products |
| myc-PICT-1-M6  1^st^ cycle PCR  2^nd^ cycle PCR | CAGGTGGCCGCTGCTGCTGCGGAGCTG  GCTGTGCACGCTGCTGCTGTACAGCAGGCC  CCGGGTACCATGGAACAAAAACTCATCTCA | CAGCTCCGCAGCAGCAGCGGCCACCTG  AACTCTAGAGCAGCTACAACTGGATCTCA  AACTCTAGAGCAGCTACAACTGGATCTCA | myc-PICT-1  myc-PICT-1-M5  1^st^ cycle PCR products |
| myc-PICT-1-del10  1^st^ cycle PCR  2^nd^ cycle PCR | CCGGGTACCATGGAACAAAAACTCATCTCA  CGGCGGCGGAGGCGGCGGCAGGCGCGG  CCGGGTACCATGGAACAAAAACTCATCTCA | CCGCCTCCGCCGCCGCTTCTCCCGCCGCCGCTG  AACTCTAGAGCAGCTACAACTGGATCTCA  AACTCTAGAGCAGCTACAACTGGATCTCA | myc-PICT-1  myc-PICT-1  1^st^ cycle PCR products |
| myc-PICT-1-del11  1^st^ cycle PCR  2^nd^ cycle PCR | CCGGGTACCATGGAACAAAAACTCATCTCA  CGGGAGGCTGAGGCTGACAAGCCCCGA  CCGGGTACCATGGAACAAAAACTCATCTCA | CTTGTCAGCCTCAGCCTCCCGCTTCTCCCGCCGCCGCTG  AACTCTAGAGCAGCTACAACTGGATCTCA  AACTCTAGAGCAGCTACAACTGGATCTCA | myc-PICT-1  myc-PICT-1  1^st^ cycle PCR products |
| myc-PICT-1-del12  1^st^ cycle PCR  2^nd^ cycle PCR | CCGGGTACCATGGAACAAAAACTCATCTCA  CGGGAGGCTGAGGCTGACAAGCCCCGA  CCGGGTACCATGGAACAAAAACTCATCTCA | CTTGTCAGCCTCAGCCTCCCGCGCCAGCTCCGCCAGCCT  AACTCTAGAGCAGCTACAACTGGATCTCA  AACTCTAGAGCAGCTACAACTGGATCTCA | myc-PICT-1  myc-PICT-1  1^st^ cycle PCR products |
| myc-PICT-1-del13 | AAAGGTACCATGGAACAAAAACTCATCTCAGAAGAAGATCTGCGGCGGCGGAGGCGGCGGCAG | AACTCTAGAGCAGCTACAACTGGATCTCA | ECFP-PICT-1 |
| myc-PICT-1-del14  1^st^ cycle PCR  2^nd^ cycle PCR | CCGGGTACCATGGAACAAAAACTCATCTCA  CAGGCACCTGACATCGACGTGCAGCTG  CCGGGTACCATGGAACAAAAACTCATCTCA | CACGTCGATGTCAGGTGCCTGCTTCTCCCGCCGCCGCTG  AACTCTAGAGCAGCTACAACTGGATCTCA  AACTCTAGAGCAGCTACAACTGGATCTCA | myc-PICT-1  myc-PICT-1  1^st^ cycle PCR products |
| myc-PICT-1-del15  1^st^ cycle PCR  2^nd^ cycle PCR | CCGGGTACCATGGAACAAAAACTCATCTCA  CCCGAGGGCAACATCCTTCGAGACCGG  CCGGGTACCATGGAACAAAAACTCATCTCA | TCGAGGGATGTTGCCCTCGGGCTTCTCCCGCCGCCGCTG  AACTCTAGAGCAGCTACAACTGGATCTCA  AACTCTAGAGCAGCTACAACTGGATCTCA | myc-PICT-1  myc-PICT-1  1^st^ cycle PCR products |
| myc-PICT-1-del16  1^st^ cycle PCR  2^nd^ cycle PCR | CCGGGTACCATGGAACAAAAACTCATCTCA  CAGAGGAGGAATATGATCGAGCCTCGA  CCGGGTACCATGGAACAAAAACTCATCTCA | CTCGATCATATTCCTCCTCTGCTTCTCCCGCCGCCGCTG  AACTCTAGAGCAGCTACAACTGGATCTCA  AACTCTAGAGCAGCTACAACTGGATCTCA | myc-PICT-1  myc-PICT-1  1^st^ cycle PCR products |
| myc-PICT-1-del17  1^st^ cycle PCR  2^nd^ cycle PCR | CCGGGTACCATGGAACAAAAACTCATCTCA  CAGAGGAGGAATATGATCGAGCCTCGA  CCGGGTACCATGGAACAAAAACTCATCTCA | CTCGATCATATTCCTCCTCTGCTGCTGCTCCGTCTTCTT  AACTCTAGAGCAGCTACAACTGGATCTCA  AACTCTAGAGCAGCTACAACTGGATCTCA | myc-PICT-1  myc-PICT-1  1^st^ cycle PCR products |
| pEGFP-PICT-1(387-478) | AAAGAATTCCGGCGGCGGAGGCGGCGGC | AAAGGATCCCTACAACTGGATCTCACG | myc-PICT-1 |
| pEGFP-PICT-1(396-478) | AAAGAATTCGAGGCTGAGGCTGACAAG | AAAGGATCCCTACAACTGGATCTCACG | myc-PICT-1 |
| pEGFP-PICT-1(387-456) | AAAGAATTCCGGCGGCGGAGGCGGCGGC | AAAGGATCCCTAGGCTCTCTCTCGAGG | myc-PICT-1 |
| pEGFP-PICT-1(396-456) | AAAGAATTCGAGGCTGAGGCTGACAAG | AAAGGATCCCTAGGCTCTCTCTCGAGG | myc-PICT-1 |
| pEGFP-PICT-1(387-412) | AAAGAATTCCGGCGGCGGAGGCGGCGGC | AAAGAATTCGGGATCAAGGCCCAGGTG | myc-PICT-1 |
| pEGFP-PICT-1(433-478) | AAAGAATTCCCCGAGGGCAACATCCTT | AAAGGATCCCTACAACTGGATCTCACG | myc-PICT-1 |
| pEGFP-PICT-1(446-478) | AAAGAATTCCAGAGGAGGAATATGATC | AAAGGATCCCTACAACTGGATCTCACG | myc-PICT-1 |
| pEGFP-PICT-1(347-386/396-478) | AAAGAATTCGCTGTGCACAGGCTGCGG | AAAGGATCCCTACAACTGGATCTCACG | myc-PICT-1-del12 |
